# Measurement of urea and creatinine in saliva of dogs: a pilot study

**DOI:** 10.1186/s12917-018-1546-5

**Published:** 2018-07-20

**Authors:** Asta Tvarijonaviciute, Luis Pardo-Marin, Fernando Tecles, Juana Dolores Carrillo, Juan Diego Garcia-Martinez, Luis Bernal, Josep Pastor, José J. Cerón, Silvia Martinez-Subiela

**Affiliations:** 1 From the Interdisciplinary Laboratory of Clinical Analysis (Interlab-UMU), Regional Campus of International Excellence “Campus Mare Nostrum”, University of Murcia, Campus of Espinardo s/n, 30100 Murcia, Spain; 2 From the Department of Animal Medicine and Surgery, School of Veterinary Medicine, University of Murcia, Murcia, Spain; 3 From the Animal Medicine and Surgery Department, School of Veterinary Medicine, University Autonomous of Barcelona, Barcelona, Spain

**Keywords:** Urea, Creatinine, Kidney, Dogs, Biofluids

## Abstract

**Background:**

Urea and creatinine in saliva have been reported to be possible markers of chronic kidney disease (CKD) in humans. The aim of this study was to assess if urea and creatinine could be measured in canine saliva, and to evaluate their possible changes in situations of CKD.

**Results:**

The spectrophotometric assays for urea and creatinine measurements in saliva of dogs showed intra- and inter-assay imprecision lower than 12% and coefficients of correlation close to 1 in linearity under dilution tests. Healthy dogs showed median salivary concentrations of urea of 39.6 mg/dL and creatinine of 0.30 mg/dL, whereas dogs with CKD showed median salivary urea of 270.1 mg/dL and creatinine of 1.86 mg/dL. Positive high correlations were found between saliva and serum activities of the two analytes (urea, *r* = 0.909; *P* < 0.001; creatinine, *r* = 0.819; *P* < 0.001).

**Conclusions:**

Urea and creatinine concentrations can be measured in canine saliva with commercially available spectrophotometric assays. Both analytes showed higher values in saliva of dogs with CKD compared with healthy dogs and their values were highly correlated with those in serum in our study conditions.

## Background

Urea and creatinine (sCr) are serum biomarkers used in dogs as endogenous indicators of glomerular filtration rate for detection of kidney disease. In addition, sCr is one of the biomarkers recommended by the International Renal Interest Society (IRIS) to evaluate and monitor renal damage/dysfunction. Both markers are currently widely used despite their limitations to detect renal disease at an early stage [1].

Saliva is a biological fluid that has various advantages as a diagnostic medium compared with blood, since its collection is non-invasive and simple, and in case of the dogs can be made by the owners. In addition, saliva can be sampled repeatedly without discomfort to the patient. In human medicine, saliva is gaining attention as a possible alternate fluid to blood analysis [2]. In dogs, analytes such as C-reactive protein [3], cortisol [4], alpha-amylase [5], adenosine deaminase [6] or muscle enzymes [7] have been successfully measured in saliva.

Previous reports in humans have found that urea and creatinine can be measured in saliva and that salivary concentrations of both analytes are positively correlated to plasma levels [8–10]. In addition, it has been suggested that analysis of salivary urea and creatinine can be used in the diagnosis of chronic kidney disease (CKD) and also for monitoring progression and efficacy of haemodialysis [11].

To the authors’ knowledge no studies about urea and creatinine in saliva of dogs have been published previously. The objective of the present study was to assess if urea and creatinine could be measured in canine saliva, and to evaluate their possible changes in situations of CKD. For this purpose, commercially available automated spectrophotometric assays for urea and creatinine measurements in saliva were validated. Moreover these analytes were measured in saliva of healthy dogs and of dogs with increased serum urea and creatinine due to CKD secondary to canine leishmaniosis.

## Material and methods

All experimental procedures were approved by the Local Ethical Committee of University of Murcia, and were performed in compliance with laws RD32/2007 and RD1201/2005 concerning animal experimentation in Spain.

### Assays

Urea was measured using a commercial kit (Beckman Coulter, Brea, USA) based on the quantification of the decrease in NADH after the hydrolysis of urea by urease. Creatinine was measured using a commercial kit (Beckman Coulter, Brea,USA) based on the Jaffe method. Specimen volume used was 2.5 μl for urea in serum and saliva and 20 μl for creatinine in serum and saliva.

All the assays were performed in an automated biochemistry analyzer (Olympus AU600, Beckman Coulter, Brea, USA) at 37 °C. Urea and creatinine assays showed an inter-assay imprecision and an inaccuracy of less than 5% in the daily quality control analysis done during the study. Manufacturer’s control solutions of two different values were used for the quality control analysis (Beckman-coulter, Lot 0037 and 0038).

### Analytical validation

For analytical validation of both methods the following parameters were calculated.

Precision. The intra-assay coefficient of variation (CV) was calculated after analysis of 2 saliva samples with different urea and creatinine concentrations 5 times in a single assay run. The inter-assay CV was determined by analyzing the same samples in 5 separate runs, carried out on different days, being the samples aliquoted and stored at − 80 °C.

Accuracy. It was evaluated indirectly by linearity under dilution. For this purpose, two canine saliva samples were serially diluted with bidistilated water.

Limit of detection. This was calculated on the basis of data from 10 replicate determinations of the zero standard (bidistilated water) as the mean value plus 3 standard deviations.

### Animals

A total of 36 dogs were included in the present study. Seventeen of these animals were healthy dogs belonging to staff and students of University of Murcia and were used as controls. None of the dogs presented abnormalities at physical and clinical examination, or in the CBC and biochemical profile, and did not have evidence of periodontal disease. Serum concentrations of urea and creatinine were lower than 50 mg/dl and 1.4 mg/dl respectively, values which represent the higher limit of the reference interval for these analytes of our laboratory.

All healthy dogs were adults with a median (range) age of 5.6 (1–11) years. Seven dogs were mongrels, five were Beagles, two were German shepherds, two were Labrador Retrievers and one was a Golden Retriever.

To evaluate if urea and creatinine can be increased in saliva from dogs with CKD, 19 animals with this disease due to canine leishmaniosis were included in the study. The dogs were naturally infected with *Leishmania infantum,* had clinical and/or laboratory test abnormalities compatible with the disease and no active sediment in urine. The dogs were classified to correspond to the group IV of the leishvet and group C of the canine leishmanosis working group [12, 13]. Leishmaniasis was diagnosed by a positive ELISA (LeiscanRLeishmania ELISA Test, Laboratorio Dr. Esteve S.A, Spain) to detect serum antibody against *Leishmania* infection and/or by visualization of *Leishmania spp*. amastigotes in bone marrow samples. This ELISA test has a high sensitivity to detect individuals harboring *Leishmania* infection [14]. All dogs showed serum concentrations of urea and creatinine higher than 50 mg/dl and 1.4 mg/dl, respectively and proteinuria (stage 4 according to the classification criteria of the International Renal Interest Society (IRIS)). In this group of dogs, age ranged between 0.9 and 12.0 years (median 6.2 years). Seven dogs were mongrels, four were German Sheperds, four were Beagles, three were Golden Retrievers and one was a Boxer.

All dogs were tested for the presence of canine heartworm, *Anaplasma phagocytophylum*, *Borrelia burgdorferi*, and *Ehrlichia canis* antibodies using SNAP test (Canine SNAP 4Dx, IDEXX Laboratories, USA) giving a negative result.

In all cases, inclusion criteria were: the saliva specimens obtained had enough volume for measurements; and no evidence of periodontal disease, since periodontitis can increase urea in human saliva [15].

### Saliva and blood sampling

The saliva and blood specimens were taken at time of diagnosis in the group of dogs with leishmaniasis.

Saliva samples were obtained by placing a sponge in dog’s mouth for 1–2 min as previously described [7]. Then the sponge was placed into the Salivette device (Salivette®, Sarstedt AG &Co., Nümbrecht, Germany) for centrifugation (P Selecta®, JP Selecta S.A, Barcelona, Spain) at 3000 x g 10 min. After saliva collection, venous blood samples (2 mL) were collected from the jugular vein into plain tubes (Vacutainer®, Plymouth, United Kingdom). Tubes were let to clot at room temperature for 30 min and centrifuged (2000 x g, 10 min) for careful removal of the serum.

The saliva and blood specimens were keep at 4 °C until being processed and measured, that were made before 1 h after collection in all cases.

### Statistical analysis

Normality of the data distribution was evaluated with a Kolmogorov-Smirnov test and, since data was not normally distributed, non-parametric tests were used. Differences in serum and salivary urea and creatinine between healthy and diseased animals were evaluated using the Mann–Whitney test. Correlations between serum and saliva urea and creatinine were calculated using the Spearman correlation test. The level of significance was set at *P* < 0.05. Statistical analyses were performed with computer software (Graph Pad Prism Version 7 for Windows, Graph Padsoftware, La Jolla, CA).

## Results

**Fig. 1:**
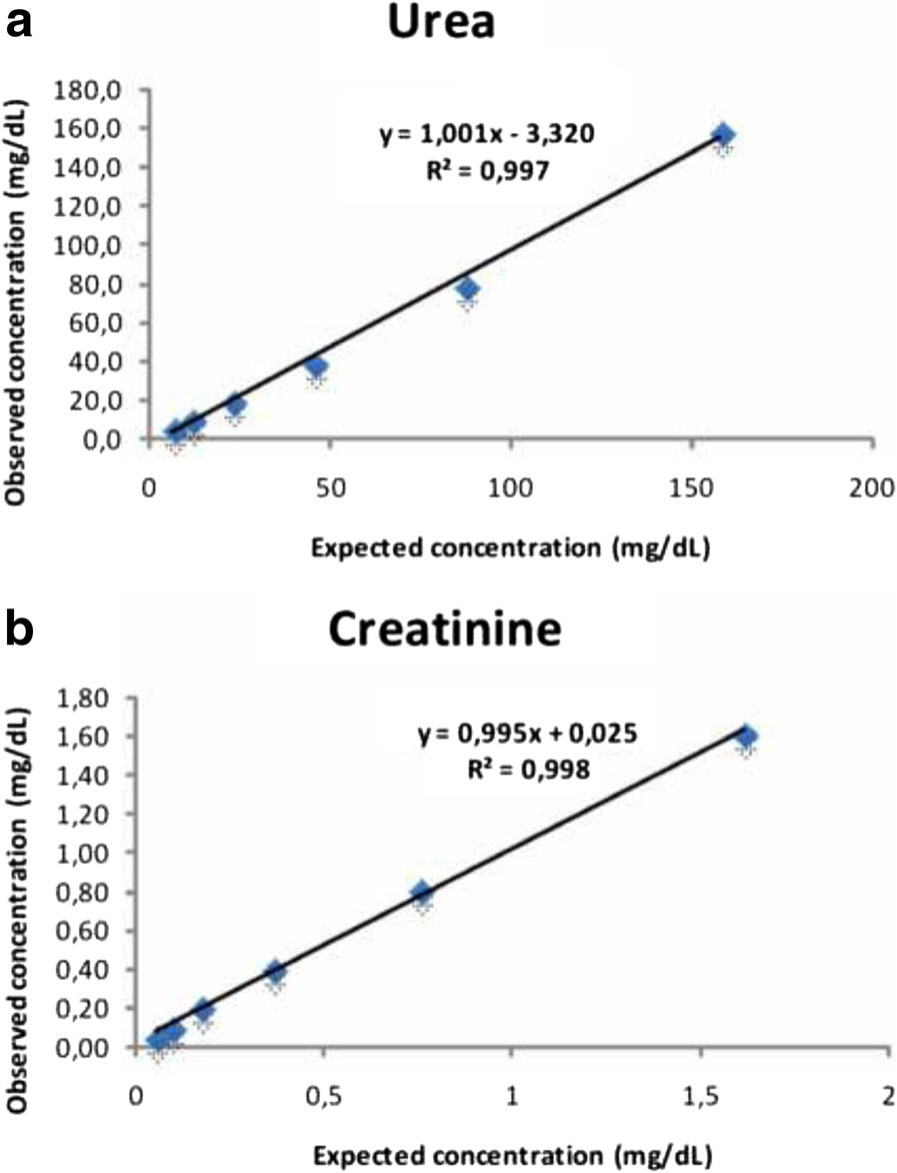
Representative graphs of linearity under dilution of saliva samples with high urea (**a**) and creatinine (**b**) concentrations

Urea in saliva showed intra- and inter-assay imprecisions lower than 2% in all cases, while creatinine in saliva showed intra- and inter-assay imprecisions lower than 9 and 12%, respectively (Table 1). Linearity under dilution resulted in coefficient of correlation close to 1 in both cases (Fig. 1). Limit of detection for salivary urea was 1.5 mg/dL (mean, 0.9 mg/dL; SD, 0.2) and for salivary creatinine was 0.03 mg/dL (mean, 0.01 mg/dL; SD, 0.007 mg/dL).

**Table 1:** Intra- and interassay coefficients of variation (CV) of urea and creatinine in canine saliva

Assay	Comparison	Sample	Mean	SD	CV (%)
Urea, mg/dL	Intra-assay	Sample 1	32.74	0.36	1.1
		Sample 2	190.84	1.04	0.5
	Inter-assay	Sample 1	32.78	0.40	1.2
		Sample 2	194.20	2.79	1.4
Creatinine, mg/dL	Intra-assay	Sample 1	0.19	0.02	8.2
		Sample 2	1.83	0.02	0.9
	Inter-assay	Sample 1	0.19	0.02	11.9
		Sample 2	1.86	0.14	7.5

**Fig. 2:**
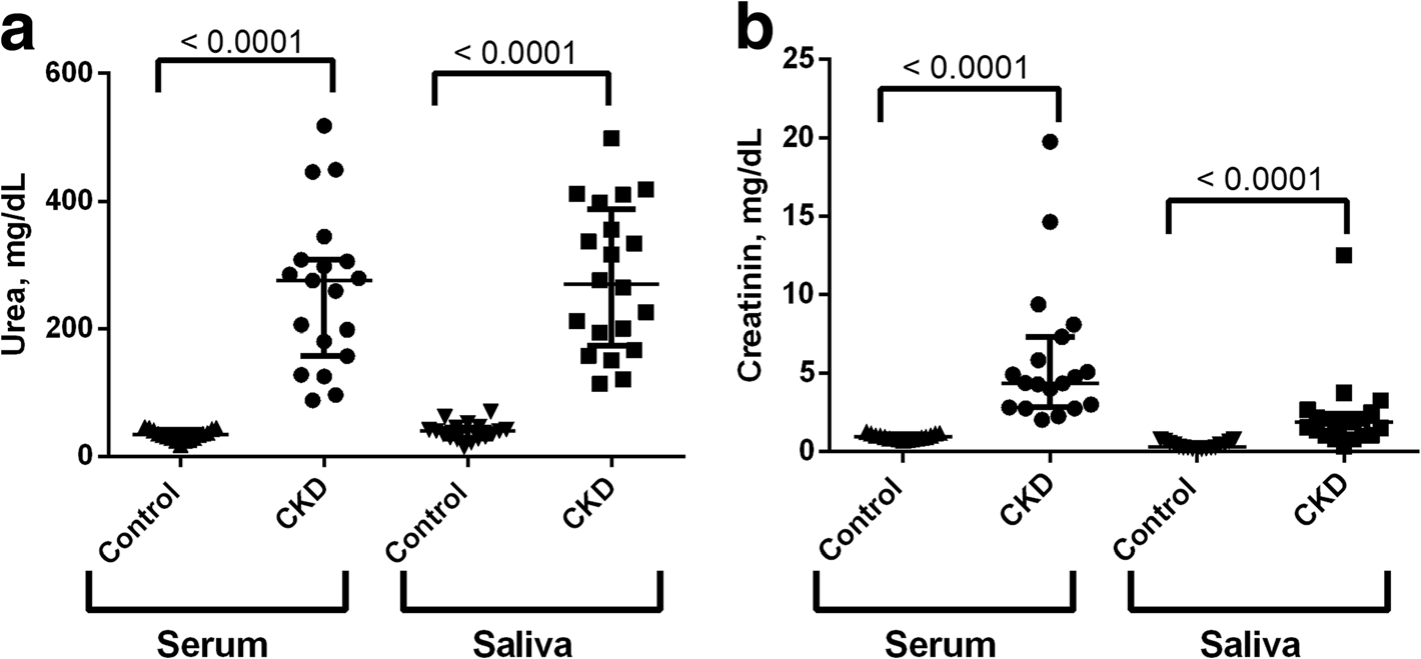
Serum and saliva urea (**a**) and creatinine (**b**) in healthy dogs (Control, *n* = 17) and dogs with chronic kidney disease (CKD; *n* = 19)

Urea and creatinine concentrations in serum and saliva in healthy dogs and dogs with CKD are shown in Fig. 2. Median (interquartile range) concentrations of urea in serum of dogs with CKD was 275.5 mg/dL (157.6–308.0 mg/dL) being higher than in healthy controls, who showed median serum urea values of 34.3 mg/dL (27.8–42.3 mg/dL) (*P* < 0.001). In saliva, the median (interquartile range) value of urea was 39.6 mg/dL (28.5–45.8 mg/dL) in healthy dogs, and in dogs with kidney failure the values were higher (*P* > 0,001) reaching a median value of 270.5 mg/dL (173.7–387.3 mg/dL). Serum creatinine concentration in healthy controls was 0.93 mg/L (0.82–1.11 mg/dL) and in dogs with kidney disease was 4.38 mg/dL (2.83–7.32 mg/dL) (*P* < 0.001). Creatinine in saliva of healthy controls was 0.30 mg/dL (0.22–0.57 mg/dL), whereas dogs with CKD showed a salivary creatinine of 1.86 mg/dL (1.04–2.42 mg/dL)(*P* < 0.001).

Spearman correlation test showed a positive correlation between serum and salivary urea (*r* = 0.909; *P* < 0.001) and between serum and salivary creatinine (*r* = 0.819; *P* < 0.001) concentrations.

## Discussion

In this paper we selected a group of dogs with CKD due to canine leishmaniosis in order to have a CKD population with a common cause, and not include the different ethiologies of CKD as a confounding factor. In dogs with leishmaniosis the CKD is produced by immune complex deposition, usually in the glomerulus, and it is a common complication of this disease [16]. Therefore, we considered this CKD population as a suitable model for evaluating our measurements of urea and creatinine in saliva.

Our study showed that salivary urea and creatinine concentrations were significantly higher in CKD dogs compared with healthy subjects. These results are in agreement with previous studies made in humans where high values of salivary urea and creatinine were observed in patients with CKD [11, 17, 18]. An explanation for these findings is that in CKD the kidneys can not excrete creatinine and urea, and therefore their blood concentrations increase leading to the diffusion of these compounds to saliva [19].

The strong correlation found in our study between serum and salivary creatinine concentrations are in line with previous reports in humans [17, 18]. Furthermore, we observed that both analytes showed a similar mean fold- increase in serum (urea, 8 fold; creatinine, 5 fold) and saliva (urea, 7 fold; creatinine, 6 fold) in dogs with CKD compared to controls. These facts could indicate that, in our study conditions, the measurements of urea and creatinine in saliva could be used as an estimation of the measures in serum, and also that urea and creatinine concentrations in saliva could be used as markers of renal failure. This would be in agreement with previous studies in humans suggesting that analysis of salivary creatinine and urea in CKD patients reflects their concentrations in blood, and that the salivary concentration of urea and creatinine could be useful in screening, diagnosis and monitoring the CKD [10, 11, 20]. In addition, this would be in line with the fact that there is a significant negative correlation between the glomerular filtration rate and salivary concentration of urea [21].

In the present study, the values of urea in serum were similar to those of saliva, both in CKD and healthy dogs. This would indicate that urea enters into saliva from blood by passive diffusion through acini of salivary glands [18]. However, creatinine in saliva showed lower values than in serum, but similar differences as serum between healthy and CKD. Low values of creatinine in saliva compared to serum have been previously reported in healthy subjects and humans with CKD [10, 17, 18]. There are various facts that could influence the lower values of creatinine in saliva, such as its large size and high molecular weight that, combined with its low lipid solubility, can limit the diffusion of creatinine across the cells and intercellular junctions of the salivary gland [17].

Although the use of saliva has many advantages such as the fact of being a non-invasive method that does not produce discomfort and pain, which can convenient in cases where dogs are reluctant to blood sampling, and the possibility of performing repeated sampling in an easy way, it has also some limitations. One is that in some cases it can be difficult to get enough volume for analysis. In addition we do not have information about how gingivitis and/or dental plaque can affect urea and creatinine concentrations in saliva of dogs and if these processes could be confounding factors. This study should be considered as a pilot one and further studies with a larger population should be performed to confirm these findings and also to evaluate the sensitivity and specificity of saliva urea and creatinine to diagnose CKD by the use of ROC curves. In addition, the ability of these tests to inform of the severity of the kidney disease and monitor the treatment, that has been demonstrated in humans [19, 20], should be tested.

For the evaluation of the specificity, it would be of interest to study salivary creatinine and urea in other different situations, such as acute kidney disease or states of dehydration, poor kidney perfusion or gastrointestinal hemorrhage that could increase the value of these analytes in serum. In addition, further studies about factors that can influence concentrations of urea and creatinine in saliva should be performed, for example age, gender, time of the day and meal. However, previous studies in humans have demonstrated that salivary urea does not show significant changes during the day, fact that could allow monitoring the conditions of patients with CKD at all time without the challenge of frequent blood sampling [9, 22]. These studies also suggested that salivary urea concentration is independent of saliva volume. In addition, the influence of different assays in creatinine measurements in saliva is a topic that would be interesting to explore, since in serum different results in creatinine measurements could be obtained depending of the method used [23].

## Conclusions

Results of the present study indicate that urea and creatinine can be measured in canine saliva with commercially available spectrophotometric assays. In addition, these analytes show higher values in saliva of dogs with chronic kidney disease compared with healthy dogs and their values have a high correlation with those of serum.
